# Anthocyanic Vacuolar Inclusions: From Biosynthesis to Storage and Possible Applications

**DOI:** 10.3389/fchem.2022.913324

**Published:** 2022-06-28

**Authors:** Kees Buhrman, Javiera Aravena-Calvo, Clara Ross Zaulich, Kasper Hinz, Tomas Laursen

**Affiliations:** ^1^ Faculty of Science, University of Amsterdam, Amsterdam, Netherlands; ^2^ Dynamic Metabolons Group, Section for Plant Biochemistry, Department for Plant and Environmental Sciences, University of Copenhagen, Copenhagen, Denmark

**Keywords:** anthocyanic vacuolar inclusions, anthocyanins, natural deep eutectic solvents, anthocyanin transport, anthocyanin storage, heterologous production

## Abstract

The ability of plants to accumulate specific metabolites in concentrations beyond their solubility in both aqueous and lipid environments remains a key question in plant biology. Natural Deep Eutectic Solvents (NADES) are mixtures of natural compounds in specific molar ratios, which interact through hydrogen bonding. This results in a viscous liquid that can solubilize high amounts of natural products while maintaining a negligible vapor pressure to prevent release of volatile compounds. While all the components are presents in plant cells, identifying experimental evidence for the occurrence of NADES phases remains a challenging quest. Accumulation of anthocyanin flavonoids in highly concentrated inclusions have been speculated to involve NADES as an inert solvent. The inherent pigment properties of anthocyanins provide an ideal system for studying the formation of NADES in a cellular environment. In this mini-review we discuss the biosynthesis of modified anthocyanins that facilitate their organization in condensates, their transport and storage as a specific type of phase separated inclusions in the vacuole, and the presence of NADES constituents as a natural solution for storing high amounts of flavonoids and other natural products. Finally, we highlight how the knowledge gathered from studying the discussed processes could be used for specific applications within synthetic biology to utilize NADES derived compartments for the production of valuable compounds where the production is challenged by poor solubility, toxic intermediates or unstable and volatile products.

## 1 Introduction

Flavonoids are aromatic specialized metabolites derived from the phenylpropanoid pathway present in plants. Anthocyanins are a subclass of flavonoids accumulating in the vacuole, giving rise to the color of most fruits, vegetables and flowers, ranging from red to purple and blue, in order to attract pollinators and seed dispersers to ensure plant reproduction (Winkel-Shirley, 2001). Additionally, anthocyanins accumulate in plant vegetative tissue, acting as photo-protection, absorbing UV light, and scavenging free radicals from PSII (Guo et al., 2008). Hence, anthocyanins and other flavonoids are of high interest as food supplements due to their antioxidant qualities promoting numerous health benefits (Konczak and Zhang, 2004; Davis et al., 2009). While their biosynthesis is well-characterized, the cellular circumstances enabling accumulation of high concentrations of anthocyanins and specialized metabolites in general remain elusive. Examples of high accumulation of specialized metabolites in plants are dhurrin in *Sorghum bicolor* (30% of dry mass) (Kojima et al., 1979; Halkier and Møller, 1989), vanillin-glucoside in *Vanilla planifolia* (Brillouet et al., 2014; Gallage et al., 2018) and anthocyanins in *Lisianthus nigrescens* (up to 24% of dry mass) (Markham et al., 2004). In *Catharanthus roseus*, flowers with Anthocyanic Vacuolar Inclusions (AVIs) have an increased concentration of anthocyanin accumulation, and exhibit a darker flower color (Markham et al., 2000; Deguchi et al., 2020). The term AVI was coined by Markham et al. (Markham et al., 2000) investigating the storage of acylated anthocyanins as inclusions in Lisianthus (*Eustoma grandiflorum*) and cyanidin and delphinidin 3,5-*O*-diglucosides in carnation (*Dyanthus caryophillus*). Following this study, presence and characteristics of AVIs were reported of in *Vitis vinifera* (Conn et al., 2003; Mizuno et al., 2006; Conn et al., 2010), rose (Gonnet, 2003), maize (Irani and Grotewold, 2005), apple (Bae et al., 2006), morning glory (Morita et al., 2005), eggplant (Umeda et al., 2006), lisianthus (Zhang et al., 2006; Chanoca et al., 2015; Kallam et al., 2017), carnation (Okamura et al., 2013), *Rhabdothamnus solandri* (Zhang H. et al., 2014), *Catharanthus roseus* (Deguchi et al., 2020), sweet potato (Zhu et al., 2018), petunia (van der Krol et al., 1993; Qi et al., 2013) and black rice (Mackon et al., 2021). Despite of the unifying AVI term, their morphology differs dramatically per plant species. The unified nature of AVIs is further challenged by the variety of experimental setups using different types of microscopy, presence of membranes around AVIs in some species, and the specific types of anthocyanin-modifications accumulating in AVIs being investigated. Moreover, few of these studies propose an extensive mechanism for the aggregation process resulting in AVIs. In this review, we summarize previously identified factors involved in AVI formation and address how the vacuolar environment and solvent characteristics have remained underexposed in studies of AVIs. Additionally, we propose a link between the formation of certain types of AVIs and the existence of a NADES phase as third intracellular phase facilitating accumulation of natural products in concentrations beyond their solubility in water and oil. The existence of such a NADES phase provides a plausible explanation for the condensation of natural products. Finally, we explore how findings on AVI formation and NADES mixtures could provide an entire new engineering avenue for improved heterologous production of flavonoids.

### 1.1 Biosynthesis of Decorated Anthocyanins Involved in Formation of Anthocyanic Vacuolar Inclusions

**FIGURE 1:**
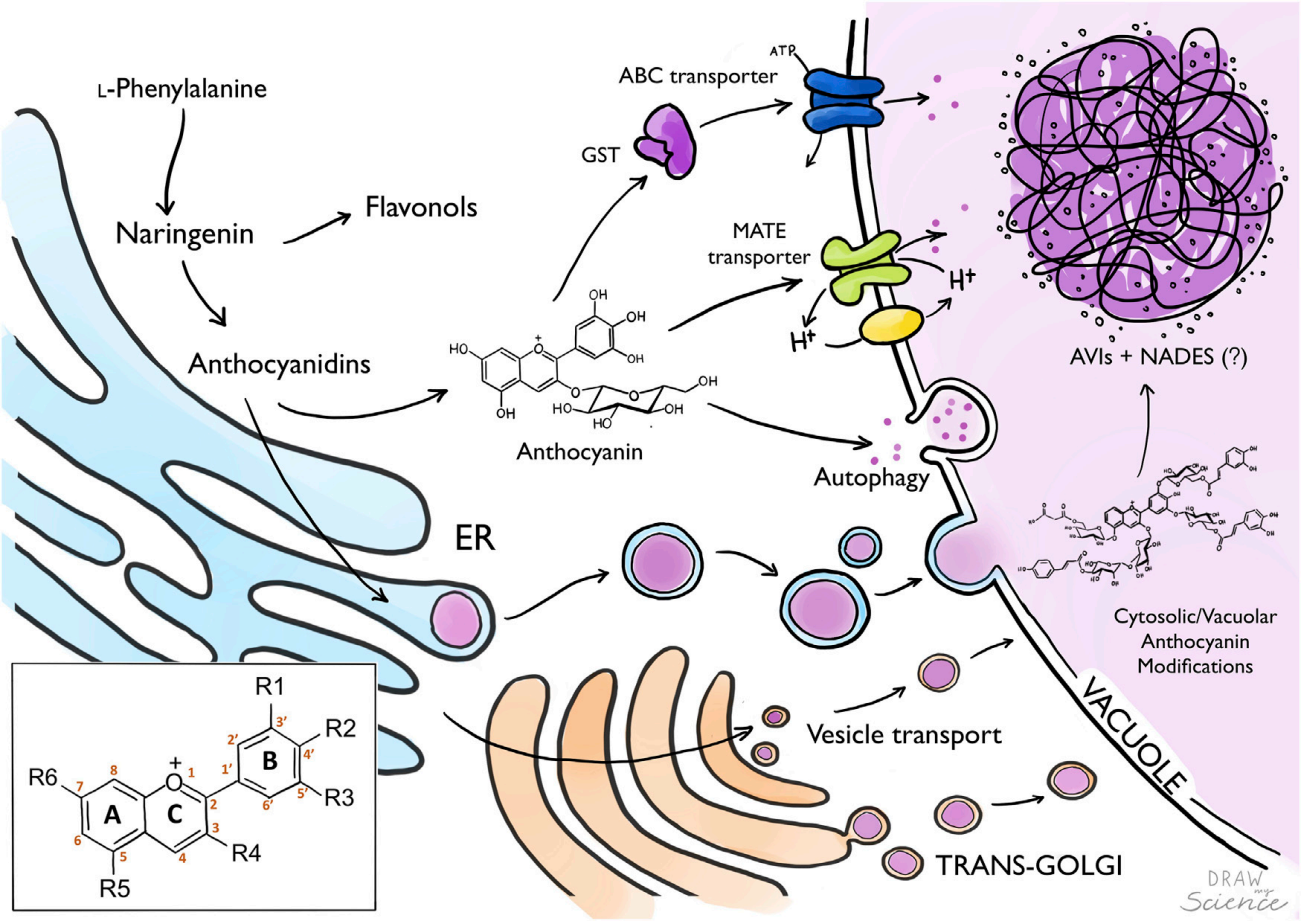
From anthocyanin biosynthesis to storage as AVIs. Biosynthesis of anthocyanins occurs in the Endoplasmatic Reticulum (ER), where anthocyanidins are later glycosylated to generate anthocyanins which are translocated to the vacuole. Anthocyanins are transported into the vacuole by tonoplast localized transport proteins (MATE, ABC-transporters) potentially mediated by Glutathione-S-Transferase. Alternatively, anthocyanins are transported into the vacuole *via* vesicles from the ER, Golgi or autophagy. Anthocyanin modification can occur in the cytosol or inside the vacuole. Formation of Anthocyanin Vacuolar Inclusions (AVIs) might be mediated by the presence of Natural Deep Eutectic Solvents (NADES) composed by primary metabolites translocated to the vacuole. Box: Anthocyanidin backbone with A, B and C ring, and six common sites for anthocyanin modifications marked with R.

The sequential steps leading to the biosynthesis of anthocyanins have been well described in the last 3 decades, and several studies indicate that this pathway is well conserved among plant species (Holton and Cornish, 1995; Winkel-Shirley, 2001; Liu et al., 2018; Yonekura-Sakakibara et al., 2019). Anthocyanins are part of the flavonoid branch derived from the core phenylpropanoid pathway where the amino acid phenylalanine is the only precursor. A key branching point toward the flavonoid pathway occurs in the conversion of coumaroyl-CoA into chalcone and naringenin, mediated by chalcone synthase (CHS) and chalcone isomerase (CHI Alternatively to anthocyanins, naringenin can be converted into flavonols by flavonol synthase (FLS) resulting in compounds such as quercetin and kaempferol, which can function as co-pigments of anthocyanins, or as floral UV-absorbing molecules attracting nocturnal pollinators (Sheehan et al., 2016). Branching out towards the anthocyanin biosynthetic pathway, flavanone-3-hydroxylase (F3H) converts naringenin to dihydrokaempferol, which is further hydroxylated in the B-ring by the action of cytochrome P450s flavonoid 3′-hydroxylase (F3′H) and flavonoid 3′,5′-hydroxylase (F3′5′H) to yield dihydroquercetin and dihydromyricetin. These three dihydroflavonols constitute the precursors that enter the final steps for anthocyanin biosynthesis. They are converted into anthocyanidins by consequently dihydroflavonol 4-reductase (DFR) and anthocyanidin synthase (ANS) yielding pelargonidin, cyanidin and delphinidin, depending on the hydroxylation of the B-ring, which affects the color significantly (Figure 1). All naturally synthesized anthocyanidins are glycosylated on the 3-position by a cytosolic glycosyltransferase to increase stability, solubility and facilitate transport to the vacuole (Zhang Y. et al., 2014; Alseekh et al., 2020). Consequently, the compounds can be subjected to various modifications on the hydroxyl groups of the backbone structure (Figure 1), such as methylation, glycosylation, and acylation. The complexity of anthocyanin decorations differs widely among plant species from simple monoglucosides to multiple substitutions of different sugars and acylations (Provenzano et al., 2014). Additionally, the color of anthocyanins is also influenced by post-biosynthetic factors. These include the pH of the vacuole (Quattrocchio et al., 2006), molecular stacking through self-association or of other co-pigments (rev. in (Zhang Y. et al., 2014; Houghton et al., 2021)), and interactions with metal ions and flavonoids or other metabolites to form metal complexes, resulting in a blue color (Yoshida et al., 2009; Schreiber et al., 2010; Schreiber et al., 2011; Ito et al., 2019). There are no studies indicating that metal-anthocyanin complexing is involved in the formation of AVIs.

Several studies have suggested a few decorations to be critical for the condensation of anthocyanins in the vacuole. A pivotal study proposed a model for molecular stacking of anthocyanins by the folding of aromatic acyl groups over the C-ring of the anthocyanin, favoring color stability and AVI formation (Kallam et al., 2017; Houghton et al., 2021). This highlighted the role of aromatic acylation in AVI formation. Constitutive expression of two transcription factors from snapdragon in tobacco for production of cyanidin 3-*O*-rutinoside, while consequent expression of a *p*-coumaroyl coenzyme A (CoA) acyltransferase from *Arabidopsis* (At3AT) resulted in AVI formation. *In-vitro*, the extracted acylated anthocyanins condensed into aggregated structures with solubility decreasing under an increasing pH. The role of aromatic acylation was supported by accumulation of AVIs in Delphinium *Morning Skies*, containing anthocyanins with four benzoyl groups, compared to the less-acylated anthocyanins in Delphinium *King Arthur* which displayed a uniform vacuolar anthocyanin distribution (Kallam et al., 2017). The importance of acylation is further supported in grape, where enrichment of acylated anthocyanins correlates with presence of AVIs (Conn et al., 2003; Mizuno et al., 2006), and *p*-coumaroylated anthocyanins in vesicle-like AVIs in *Arabidopsis*. However, AVIs do not only accumulate when anthocyanins are aromatically acylated, as in Carnation AVIs accumulate in mutants unable to malonylate (Okamura et al., 2013), and in *Rhabdothamnus solandri* AVIs contain simpler anthocyanin-3-*O*-glucoside species (Zhang H. et al., 2014). Additionally, a *5gt* mutant unable to glycosylate at the 5-position exhibited an increased accumulation of AVIs (Pourcel et al., 2010). An NMR study showed that purified anthocyanin-3-*O*-glucosides and coumaroylated 3-*O*-glucoside anthocyanins self-associate *in-vitro* (Fernandes et al., 2015) which could be an indication why in different plant species AVIs are enriched in these type of anthocyanins. Based on thermodynamic analysis both methylation and hydroxylation of the anthocyanin B-ring was suggested to favor condensation (Leydet et al., 2012). However, overexpression of a F3′5′H from petunia in the tobacco cells accumulating cyanidin 3-*O*-rutinoside resulted in production of delphinidin 3-*O*-rutinoside but no AVI formation (Kallam et al., 2017). In contrast, hydroxylation of the anthocyanin B-ring in *Petunia* by constitutive expression of F3′5′H from *Phalaenopsis* (orchid) resulted in accumulation of delphinidin and formation of visible AVIs (Qi et al., 2013). There is clear indication of aromatic acylation playing an important role in AVI formation. However, not in all AVIs, which allows for speculation on other factors involved in AVI formation.

### 1.2 Molecular Environment Facilitating Anthocyanic Vacuolar Inclusions Formation

**FIGURE 2:**
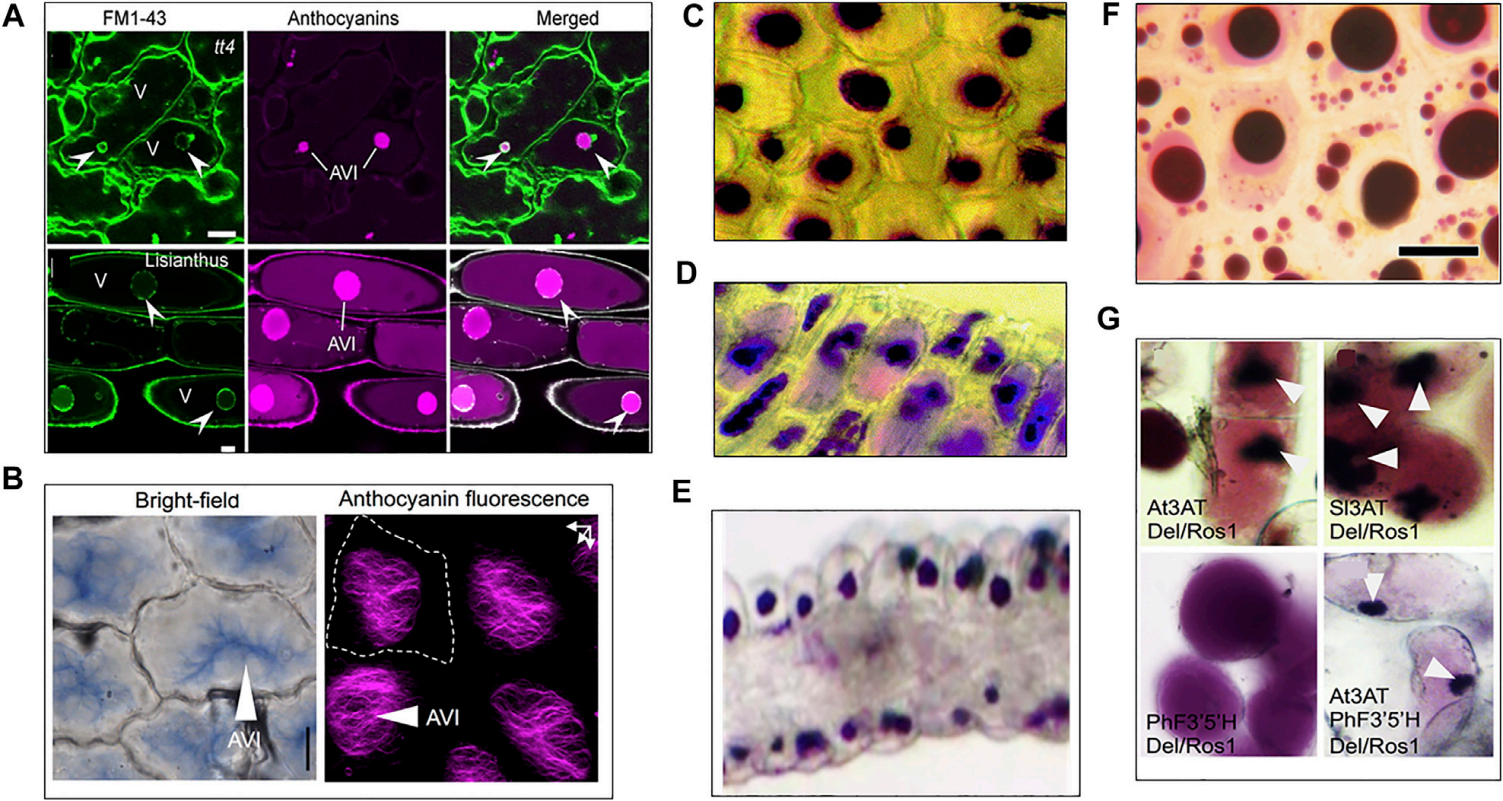
Diversity of AVI morphology across plant species. **(A)** Confocal micrographs of membrane bound AVIs in Arabidopsis (top, *tt4*) and Lisianthus (bottom), membrane fluorescently marked with FM1-43 dye. (Chanoca et al., 2015). **(B)** AVIs found in *Delphinium grandiflorium* sepal cells (Kallam et al., 2017). **(C)** AVIs in carnation (Markham et al., 2000) **(D)** AVIs in Lisianthus (Markham et al., 2000). **(E)** granular AVIs in *Catharanthus roseus* (Deguchi et al., 2020). **(F)** Vesicle-shaped AVIs enriched in acylated anthocyanins visible in grapevine cells (Mizuno et al., 2006). **(G)** AVIs formation in tobacco after aromatic acylation of anthocyanins: top left and right, AVIs visible in tobacco callus expressing Anthocyanin inducing transcription factors and acyl transferases from *Arabidopsis* and tomato (AT3AT and Sl3AT). Bottom left, tobacco callus expressing anthocyanin inducing transcription factores and *Petunia* Flavonoid-3′5′-Hydroxylase and shows no AVIs. Bottom right, tobacco callus expressing anthocyanin inducing transcription factors, *Arabidopsis* acyl transferase and *Petunia* Flavonoid-3′5′-Hydroxylase accumulates AVIs (Kallam et al., 2017).

When comparing the morphology of AVIs from different species (Figure 2), it is remarkable to see that they tend to group into a limited subset of structural determinants. Most AVIs are described as vesicle-like structures, where others have a round but more granular morphology. In Arabidopsis and Lisianthus, vesicle-shaped AVIs were enveloped by a membrane (Figure 2 (Chanoca et al., 2015)), making it likely similarly shaped AVIs have a membrane as well. The absence of a membrane around granular AVIs in Lisianthus and Tobacco indicates it may be common for similarly shaped AVIs to be membrane-less (Markham et al., 2000; Kallam et al., 2017). Interestingly, the morphology of AVIs in *Delphinium grandiflorum* is radically different (Figure 2 (Kallam et al., 2017)), as they form a type of filamentous crystalline shape. Moreover, confocal micrographs of the AVIs and FM1-43 stained tissue seem to indicate an additional membrane-less separation of the region containing the AVI from the rest of the vacuole (Kallam et al., 2017). Besides the actual flavonoid composition inside the vacuole, the molecular environment facilitating anthocyanin condensation remains unexplored.

#### 1.2.1 Proteinaceous Membraneless Compartments

Protein guided liquid-liquid phase separation is known from a variety of molecular processes providing membraneless sub-compartments preventing bulk equilibria (Banani et al., 2017; Zheng et al., 2021). The presence of disordered regions or entirely unstructured peptides facilitate such phase separation processes and may be a key driver for AVI formation. The presence of proteins in or around AVIs has been reported in Lisianthus (Markham et al., 2000) and sweet potato (Nozue et al., 1995; Nozue et al., 1997; Xu et al., 2001; Nozue et al., 2003). Markham et al. describe finding “proteinaceous” material in Lisianthus inclusions, however, there have not been any follow-up publications on the exact type of protein present in AVIs in these flowers (Markham et al., 2000). In sweet potato, expression of a vacuolar protein (VP24) correlated with the accumulation of anthocyanins in vacuoles, and immunocytochemical detection showed the protein co-localized with AVIs (Nozue et al., 1995). A proteomics study underlined accumulation of VP24 in purple sweet potato, and proposed the protein could be involved in the degradation of anthocyanin-glutathione, although anthocyanin-glutathione conjugates have never been observed. The authors also propose that VCaB42 and VP24 together mediate microautophagy resulting in anthocyanin transport and membrane-bound AVI formation (Wang S. et al., 2016).

#### 1.2.2 Natural Deep Eutectics Solvents and the Vacuolar Environment of Anthocyanic Vacuolar Inclusions

Beyond oil and water, Natural Deep Eutectic Solvents (NADES) may provide a third intracellular phase composed of stoichiometric mixtures of common general metabolites, such as amino acids, organic acids, sugars, and choline (Choi et al., 2011). Inspired by nature, the high solubility of specialized metabolites in NADES is being promoted as a “green” alternative to commonly used organic solvents for extraction of a broad diversity of specialized metabolites including the flavonols quercetin and kaempferol, and anthocyanins (Dai et al., 2013a; Dai et al., 2013b; Vanda et al., 2018). Moreover, enzymes of the biosynthetic pathway of dhurrin were able to retain activity after incubation at various temperatures when incubated in a NADES mixture of glucose and tartrate, as opposed to glycerol or water (Knudsen et al., 2020). Additionally, enzymes in NADES mixtures have shown increased activity (Milano et al., 2017; Elgharbawy et al., 2018; Elgharbawy et al., 2020; Panić et al., 2021; Thomas and Kayser, 2022). The increased stability and activity of enzymes, high solubility of specialized metabolites, and omnipresence of the constituents of NADES mixtures in all types of organisms are indications that these types of mixtures could constitute an important aspect of the natural environment in cells. However, *in-vivo* proof of NADES mediated liquid-liquid phase-separation is still lacking. Although experimental proof is missing, the tonoplast membrane surrounding the vacuole contains a plethora of general metabolite transporters involved in the homeostasis of the cytosol (rev. in (Martinoia et al., 2012)), allowing vacuolar accumulation of general metabolites (Davies et al., 2006). Therefore, common NADES constituents such as organic acids and sugars can accumulate in high concentrations in the vacuole (Schulze et al., 2002; Lecourieux et al., 2014). The pioneering work proposing a biological function of a NADES phase in plant cells was based on analyses of general metabolites and the interaction between sucrose and malic acid into liquid crystals using NMR (Kim et al., 2010; Choi et al., 2011). Similar NMR methods could be applied on species accumulating high concentrations of specialized metabolites to identify NADES mixtures involved in their solubilization. The development of mass spectrometry imaging with subcellular resolution (Cornett et al., 2007; Bjarnholt et al., 2014) can provide a key approach to experimentally validate the existence of NADES sub-compartments. Such compartments could easily be co-localized with anthocyanins based on the inherent pigment properties. Additionally, micro-syringe or laser dissection to harvest sub-compartments facilitated by NADES mixtures could be an option to isolate and identify the metabolites involved in the solubilization, as has been proposed in a previous review on NADES and natural products (Møller and Laursen, 2021).

### 1.3 The Anthocyanin Traffic Routes From Biosynthesis to Anthocyanic Vacuolar Inclusions

Although biosynthesis occurs at the ER, the last decorations of anthocyanins and other specialized metabolites such as glycosylation and acylation may actually occur in the vacuole, using different types of acyl-sugars as donors (Matsuba et al., 2010; Sasaki et al., 2014; Orme et al., 2019; Yonekura-Sakakibara et al., 2019). This indicates that specific “mature” anthocyanins are selectively transported from the ER to the vacuole where the final decorations may result in their condensation into AVIs. Furthermore, this would provide a means to prevent aggregation in the cytosol. Multiple studies have been published on anthocyanin transport, yet still there is no consensus on the exact mode of transport (Braidot et al., 2008; Grotewold and Davies, 2008; Petrussa et al., 2013; Biała and Jasiński, 2018; Pečenková et al., 2018; Kaur et al., 2021). Most likely, anthocyanin transport may involve multiple routes depending on the plant, tissue and developmental stage, and perhaps the type of structural anthocyanin modification. One model proposes anthocyanins to be transported in vesicles from the ER to the vacuole, whereas the other model proposes a combined effort of cytoplasmic and tonoplast localized transporter proteins (Figure 1) (Zhao and Dixon, 2010). These models are not mutually exclusive.

#### 1.3.1 Selective Vacuolar Loading of Anthocyanins Governed by Transport Mechanism

Two key transporters are involved in active transport of mature anthocyanins across the tonoplast membrane. MATE-transporter proteins belong to the multidrug/oligosaccharidyl-lipid/polysaccharide (MOP) superfamily. These transporters have been shown to transport acylated anthocyanins in grape (Gomez et al., 2011), and malonylated anthocyanins in legume (Zhao et al., 2011). MATE-transporters use the electrochemical gradient of protons formed by tonoplast localized V-ATPases and a H^+^-pyrophosphatase for secondary active transport (Gaxiola et al., 2007; Gomez et al., 2009). ABC-transporters are involved in the transportation of glycosylated anthocyanins to the vacuole (Goodman et al., 2004; Francisco et al., 2013; Behrens et al., 2019). These transporters are dependent on reduced glutathione (GSH) provided by a glutathione-S-transferase (GST). Genes encoding GSTs have been frequently linked to anthocyanin transport and have been identified in many different plants such as *Petunia* (*AN9*), *Arabidopsis* (*TT19*), and grape (*VvGST1* and *VvGST4*) (Alfenito et al., 1998; Mueller et al., 2000; Kitamura et al., 2004; Conn et al., 2010). Usually, GSTs form conjugates with their substrate, to facilitate transport across the tonoplast; however, anthocyanin-GSH conjugates have never been found (Mueller et al., 2000). The GST is therefore hypothesized to simply bind the anthocyanin and “escort” it to the transporter localized in the tonoplast with GSH. However, in *Arabidopsis* grown under conditions to induce anthocyanin accumulation (Poustka et al., 2007; Pourcel et al., 2010; Chanoca et al., 2015), the prevalence of AVIs increased when ABC-transporters were inhibited, as well as under glutathione depletion (Poustka et al., 2007). Additionally, *tt19* knock-out plants exhibited cytoplasmic anthocyanin aggregates, as well as AVIs, but little soluble vacuolar anthocyanins (Chanoca et al., 2015).

Alternative to active transport mediated by tonoplast localized transporters, anthocyanins may enter the vacuole via vesicle mediated transport (Zhang et al., 2006; Sun et al., 2012). In this route, flavonoids synthesized on the cytosolic site of the ER are transported to the lumen of the ER where they accumulate into vesicle-like structures (Figure 1). These structures have been observed to be associated with the formation of AVIs in the vacuole (Zhang et al., 2006; Conn et al., 2010). While these vesicles are visible by microscopy techniques, the evidence of canonical vesicle transport proteins such as cargo, GTPases, VSRs and SNAREs are still lacking (Conn et al., 2010; Zhao and Dixon, 2010). Therefore, it is hypothesized the anthocyanin vesicles are engulfed by autophagosomes and delivered to the vacuole by autophagy (macro and microautophagy) and stored as membrane surrounded AVIs (Figure 2) (Pourcel et al., 2010; Chanoca et al., 2015). An extensive study on AVI formation and trafficking showed that AVIs formed after microautophagy, and that anthocyanins in the ER did not aggregate (Chanoca et al., 2015). This may imply the importance of vacuole-localised modifications of anthocyanins and their transport to the vacuole for the formation of anthocyanin biocondensates, which could be of value for successful heterologous synthesis of flavonoids.

## 2 Discussion

Like many biological systems, the formation of AVIs is highly complex and may involve multiple processes ultimately leading to a variety of morphological distinct anthocyanin condensates in the vacuole. Here we highlight that hydroxylation of the B-ring, 3-*O*-glycosylation and aromatic acylation may be key for molecular stacking and condensation. The condensation process appears to happen in the vacuole potentially driven by further modifications catalyzed by vacuolar transferases. Some AVIs are membrane-less and typically appear grainy in structure whereas a membrane surrounds others, which display liquid droplet-like behavior such as fusion and homogeneous distribution of the anthocyanins (Figure 2). This morphology may be linked to the type of transport (Figure 1). In previous studies on AVI formation, the nature of the vacuolar environment of AVIs, and its role in AVI formation has remained underexposed. Experimental investigation of the vacuolar environment of AVIs is required to characterize the molecular environment governing formation of AVIs. Based on the *in vitro* effect of NADES mixture on enzyme stability and productivity, and the extraordinary solvent properties, we propose that NADES could provide the molecular environment in (plant) cells allowing high catalytic efficiency of enzymes, and high accumulation of specialized metabolites.

### 2.1 Synthetic Vacuolar Inclusions for Heterologous Production of Complex Flavonoids

Several flavonoids are in clinical trials as a potential treatment against life-style and aging induced chronic inflammation (Ginwala et al., 2019) and they are of interest as natural food colorants to replace synthetic dyes (Oplatowska-Stachowiak and Elliott, 2017). The bioactivity of these molecules depends on highly specific decorations and they are typically present in minute amounts in the natural plant sources. Synthesizing flavonoids in a sustainable and cost-efficient way fits in an agenda promoting a switch to sustainable circular synthesis of natural products (Sørensen et al., 2022), harnessing the ability of plants, as exhibited in apple calli (Wang N. et al., 2016) and tobacco cell culture (Appelhagen et al., 2018), and scaling it to a larger scale. Plant cell culture (Appelhagen et al., 2018) or microbial micro-factories both constitute promising sustainable ways of anthocyanin synthesis for commercial purposes. Plant cell culture benefits from the native presence of flavonoid and anthocyanin biosynthetic pathways in plants, where the overexpression of specific transcription factors would lead to the activation of these pathways. (Appelhagen et al., 2018). However, biosynthesis of more specific and modified anthocyanins would require many more steps to be introduced, which minimizes the initial benefit of a system activated by transcription factors. On the other hand, while heterologous synthesis of anthocyanins in microbes would require the introduction of entire pathways, they are cheaper to use, and have been heavily optimized for heterologous production of highly complex plant natural products (Luo et al., 2019; Srinivasan and Smolke, 2020) and therefore offer a system, that is, easier to scale-up. Within the last decades, studies have reported heterologous production of anthocyanins in both *Escherichia coli* and *Saccharomyces cerevisiae* [rev. in (Sunil and Shetty, 2022)]. A recurring problem during heterologous expression of biosynthetic pathways in microbial cells is secretion into the growth-media before the finalized product is synthesized, resulting in a mixture of intermediates and products. The knowledge gathered on biosynthesis, transport and storage of anthocyanins in plants could prove essential for the quest to engineer the next generation of micro factories for heterologous production of flavonoids in the vacuole of, e.g., yeast cells. Condensation into Synthetic Vacuolar Inclusions (SVIs) by targeted modifications in combination with directed transport within the yeast cells would prevent auto-toxicity and enable accumulation of molar concentrations of metabolites, as demonstrated in plant cells for storage of vanillin (Brillouet et al., 2014; Gallage et al., 2018), dhurrin (Kojima et al., 1979; Halkier and Møller, 1989), and anthocyanins (Markham et al., 2004). Like organisms from other kingdoms, yeast accumulates general metabolites required for formation of a NADES phase (Choi et al., 2011). Therefore, the *in-vivo* application of NADES derived SVIs in yeast cells might simply be achieved by introducing a transport system designed by plants for selective transport of anthocyanins with proper decorations required for condensation. Overall, this could present an entirely new approach to avoid auto-toxicity, leakage of non-decorated compounds and prevent cross-talk with native pathways. In summary, the importance of NADES in AVI formation remains a topic in need of further research which may become an essential stepping stone for future production of flavonoids in heterologous systems.
